# Histologic Transformation in Follicular Lymphoma: Real-World Outcomes with Rituximab vs. Obinutuzumab-Based Combinations

**DOI:** 10.3390/cancers18091471

**Published:** 2026-05-03

**Authors:** Dor Shpitzer, Chava Perry, Tamir Shragai, Guy Melamed, Mitchell R. Smith, Roy Vitkon, Hillel Alapi, Irit Avivi

**Affiliations:** 1 Department of Haematology, Tel-Aviv Sourasky Medical Center, Tel-Aviv 6423906, Israel; chavap@tlvmc.gov.il (C.P.); tamirsh@tlvmc.gov.il (T.S.); royv@tlvmc.gov.il (R.V.); iritavi@tlvmc.gov.il (I.A.); 2 Gray Faculty of Medical and Health Sciences, Tel Aviv University, Tel Aviv 6997801, Israel; melamed_gu@mac.org.il; 3 Kahn Sagol Maccabi Research & Innovation Center, Maccabi Healthcare Services, Tel Aviv 6812509, Israel; alapi_h@mac.org.il; 4 Follicular Lymphoma Foundation, London NW3 6HJ, UK; mitchell@theflf.org

**Keywords:** follicular lymphoma, obinutuzumab, real-world study

## Abstract

**Background/Objectives**: The survival of patients experiencing transformation of follicular lymphoma (tFL) is generally inferior to that of their non-transformed FL counterparts. While rituximab (R) has been shown to reduce transformation rates, data on Obinutuzumab (O), a third generation anti-CD20 monoclonal antibody, are limited. **Methods**: This retrospective study analyzed risk factors for tFL and evaluated outcomes following transformation in 1145 consecutive patients with FL (2010–2023). **Results**: Over a median follow-up of 70 months, 9% (n = 103) of FL patients developed tFL, with a median time of 36 months from FL diagnosis to transformation. In multivariate analysis, O-based, compared to R-based regimens and maintenance therapy (compared to no maintenance), were independently associated with reduced risk of histologic transformation (HR 0.40, 95% CI 0.19–0.90, *p* = 0.026 and HR 0.42, 95% CI 0.23–0.77, *p* = 0.005, respectively). Transformed FL was associated with shorter overall survival (OS) from FL diagnosis compared to non-transformed FL (median not reached, *p* < 0.001), while prior exposure to anti-FL chemoimmunotherapy predicted shorter OS following transformation (median 34.6 months vs. not reached, *p* = 0.001). Multivariate analysis confirmed prior exposure to anti-FL therapy (HR 5.27, *p* < 0.001), male sex (HR 2.36, *p* = 0.014), and age over 65 (HR 2.54, *p* = 0.019) to be associated with shorter OS among patients with tFL. **Conclusions**: These findings, although requiring further validation, suggest that O-based regimens may reduce the risk of transformation and support prior studies demonstrating adverse survival outcomes in previously treated tFL patients.

## Simple Summary

Follicular lymphoma (FL) is a prevalent hematologic malignancy characterized by its potential for histological transformation into an aggressive lymphoma, which significantly compromises patients’ prognoses. Identifying predictors of transformation while applying modern therapeutic strategies to mitigate this risk are essential for optimizing clinical management. In this large-scale, real-world cohort study, we evaluated the incidence and kinetics of transformation alongside the impact of various frontline treatments. Our findings demonstrate that obinutuzumab-based regimens and maintenance therapy are independently associated with a reduced risk of transformation. Conversely, patients who received prior systemic therapy before undergoing transformation experienced inferior survival outcomes. These data provide real-world insights that support personalized therapeutic strategies.

## 1. Introduction

Transformation of follicular lymphoma (FL) into a more aggressive histology, most commonly diffuse large B-cell lymphoma (DLBCL), remains a critical clinical event associated with significantly reduced survival [1,2,3]. The annual risk of histologic transformation (HT) is estimated at approximately 1–2%. The cumulative incidence rates reach 8–16% at 10 years post-diagnosis, and the highest risk is observed within the first 2 to 5 years following initial diagnosis [4,5,6,7,8,9,10,11,12]. Large population-based studies, such as the National LymphoCare Study (NLCS), have reported even higher rates of HT (up to 14.3% over a median follow-up of 6.8 years), particularly among patients presenting with advanced-stage disease [9,13,14]. Although the advent of anti-CD20 monoclonal antibodies has substantially diminished the risk of transformation (cumulative 10-year hazard estimated as 5.9% in patients treated with R induction and 3.6% in patients treated with R maintenance) [5], transformed FL (tFL) continues to confer poor prognosis [4,7,8,9]. Early transformation-defined as occurring within 24 months of FL diagnosis and prior exposure to chemoimmunotherapy are both strong predictors of inferior outcomes, with two-year overall survival (OS) rates declining to 46.8% and 35–40%, respectively [4,15,16].

Despite advances in frontline therapeutic strategies, including O-based regimens, the real-world impact of these novel approaches on the risk of HT in patients with FL remains uncertain. The GALLIUM trial, a phase 3 study, demonstrated that O in combination with chemotherapy (such as bendamustine) reduced early progression, prolonged progression-free survival, and deepened remissions compared to R-based regimens. It also showed a tendency towards decreased incidence of tFL among patients treated with O compared to R [4]. However, the reported findings regarding the effect of O on HT have yet to be robustly validated in routine clinical practice. Furthermore, the effect of maintenance therapy on mitigating the risk of transformation remains controversial, with conflicting data across studies [7,9].

This study aims to address these gaps by providing real-world data on the incidence and timing of tFL in patients with FL, evaluating the association between first-line and maintenance therapies and the risk of transformation, as well as identifying clinical predictors of OS following transformation.

## 2. Materials and Methods

### 2.1. Study Design and Data Source

This research is a non-interventional, retrospective observational cohort based on anonymized data derived from the electronic medical records of Maccabi Healthcare Services (MHS) members. MHS is a nationwide healthcare organization, serving a quarter of the population in Israel. The study was approved by the institutional ethics committee (approval number MHS-0075-23).

The MHS electronic medical database contains longitudinal data on a stable population of 2.8 million people and possess several automatically formulated registries. Data for our study were automatically collected and included laboratory results from a single central lab, as well as detailed demographic and clinical information.

### 2.2. Study Population

We identified all consecutive patients diagnosed with FL grade 1, 2, 3A (ICD-9 codes 202.0–202.1) between January 2010 and December 2023. A total of 1145 patients with a confirmed FL diagnosis were included. Inclusion criteria were age ≥ 18 years at the time of FL diagnosis and continuous registration with MHS for at least 12 months prior and after diagnosis. tFL was defined as a recorded diagnosis of a biopsy-proven HT to DLBCL in the medical records (ICD-9 codes 200.7–200.78), documented in medical notes of patients with prior diagnosis of follicular lymphoma (low grade). ICD codes were recorded only in cases with a confirmed histological diagnosis. Patients who had a diagnosis of DLBCL/FL grade 3B prior to or within three months of the diagnosis of FL were excluded. This criterion was established to ensure the study captured true histologic transformation and omitted cases of transformed or composite disease present at baseline.

### 2.3. Study Variables and Definitions

Baseline characteristics at the time of FL diagnosis included demographic information, comorbid conditions, and laboratory parameters. Socioeconomic level was derived from an income index categorized into national deciles (1 = lowest income, 10 = highest). Recorded pre-existing comorbidities were non-insulin-dependent diabetes mellitus (NIDDM), chronic kidney disease (CKD), cerebrovascular accident (CVA), congestive heart failure (CHF), hypertension (HTN) and a history of cancer (excluding non-melanoma skin cancer). Laboratory values recorded at diagnosis were hemoglobin (Hb), lactate dehydrogenase (LDH), and beta-2 microglobulin (B2M), all obtained from MHS central laboratory data [17,18,19].

Charlson Comorbidity Index (CCI) scores were calculated using International Classification of Diseases (ninth revision) coding system [20]. Anemia was defined as Hb < 12 g/dL, elevated LDH as >280 U/L (the upper normal range) and elevated B2M as >3 mg/L.

First-line treatments were documented and classified into the following categories: rituximab (R) combined with cyclophosphamide, doxorubicin, vincristine, and prednisone (R-CHOP) or with cyclophosphamide, vincristine and prednisone (R-CVP); R plus bendamustine (BR); R monotherapy; obinutuzumab (O) combined with CHOP/CVP or O combined with B (OB).

The distribution of treatment regimens reflected national treatment policies and routine clinical practice in Israel during the study period. (Bendamustine became available in Israel in 2012 and has been reimbursed as first-line therapy since 2015. Obinutuzumab was reimbursed as first-line therapy in FL in 2018). Anti-FL therapies were identified based on documented medication purchases.

Maintenance therapy was initiated approximately 8 weeks following the final induction cycle and was analyzed as a fixed variable (exposure vs. no exposure) [7].

Patients were categorized into three groups: (I) Upfront treated cohort—patients who initiated anti-FL chemoimmunotherapy within three months from diagnosis of FL; (II) Deferred treatment cohort—patients who were initially managed with observation but subsequently required anti-FL chemoimmunotherapy, initiated beyond three months from diagnosis of FL; and (III) Treatment-naïve cohort—patients who did not receive chemoimmunotherapy treatment during the entire study period (in non-transformed patients) or prior to transformation (in tFL). Patients who were treated solely with local radiotherapy were included in this cohort.

FL and DLBCL diagnoses were based on ICD-9 codes as detailed above. The date of the first documented DLBCL diagnosis was defined as the transformation event. Outcomes assessed included time to transformation and OS. Factors associated with increased risk of transformation and inferior survival post-transformation were assessed.

### 2.4. Statistical Analysis

Descriptive statistics were presented as absolute numbers and percentages for categorical variables and as means or medians with standard deviations for continuous variables.

The Chi-squared test and Mann–Whitney test were used for comparisons of proportions and medians across groups, respectively. All tests were two-tailed, and 0.05 level was used for significance. Censoring was defined as the last known follow-up date within the MHS system. Patients were followed from diagnosis until death or until the end of the study period, whichever occurred first.

Fine–Gray competing risk analysis was used to estimate the cumulative transformation rate, with “death” regarded as a competing risk. Kaplan–Meier method was used to generate survival curves, presented as transformation-free survival and OS. Log-rank test was performed to assess differences in the survival curves between groups. Univariate and multivariate Cox proportional hazards regression models were used to obtain hazard ratios and adjusted hazard ratios, respectively, for risk of transformation and overall mortality. All analyses were conducted using the R statistical computing software (version 4.4.1) [21].

## 3. Results

### 3.1. Patient Characteristics

A total of 1145 FL patients were included in the analysis, of whom 47% (n = 543) were males, with a median age of 61 years (range 19–93) at the time of diagnosis of FL. During a median follow-up of 70 months, 9% of the patients (n = 103) had documented transformation to DLBCL (tFL).

There were no significant differences between patients who developed tFL and those who did not in terms of gender distribution (48% vs. 47% male, respectively; *p* > 0.9) or age at diagnosis (median age 63 vs. 61 years, *p* = 0.1). Comorbidities were similarly distributed between the groups, with comparable rates of NIDDM (17% vs. 21%, *p* = 0.3), HTN (42% vs. 38%, *p* = 0.4), and advanced CKD (1% vs. 1.6%, *p* > 0.9). However, tFL patients had a higher median Charlson Comorbidity Index (CCI): 4 vs. 3 (*p* = 0.007), indicating a greater burden of additional medical conditions (Table 1).

### 3.2. Patient Management at FL Diagnosis

There were no statistically significant differences in patients’ management at the time of FL diagnosis between patients with tFL and patients with non-transformed FL: 41% (n = 42) of patients with tFL and 36% (n = 373) of the patients with non-transformed FL received upfront chemoimmunotherapy, 51% (n = 53) and 58% (n = 601) were initially managed with observation, and 8% and 6% (n = 8, n = 68) were initially treated with local radiotherapy. Among all patients that were initially managed with observation or local radiotherapy, 33% (n = 244) subsequently required additional chemoimmunotherapy during the FU period.

### 3.3. Treatment Regimens

Of the 620 patients who were treated with first-line chemoimmunotherapy regimens during the study period and before tFL, 21% (n = 133) were treated with OB, 9% (n = 54) were treated with O-CHOP/CVP, 29% (n = 179) with BR, 30% (n = 183) with R-CHOP/RCVP and 11% (n = 71) with R monotherapy. Notable temporal differences were observed in first-line treatment patterns, with R-CHOP/CVP regimens predominating during 2010–2013 (70%), and increased use in BR and OB regimens (after their inclusion in national reimbursement policies) in the following years. These regimens accounted for 59% of first-line treatments in 2018–2021 and 61% in 2022–2024 (Figure S1). Consistent with these trends, the follow-up duration for patients receiving O-based regimens was shorter than for those receiving R-based therapy (median 48 vs. 86 months, *p* < 0.001). (Table S1 compares the characteristics of patients treated with R-based regimens, O-based regimens and patients that did not receive treatment).

### 3.4. FL Treatment in Patients with tFL

Among the 103 patients with tFL, 47% (n = 49) were treatment-naïve at the time of transformation, whereas 53% (n = 54) had previously received chemoimmunotherapy. Of these 54 pre-treated patients, 67% (n = 36) received an upfront chemoimmunotherapy, while 33% (n = 18) were initially management with observation (26%, n = 14) or local radiotherapy (7%, n = 4) and received chemoimmunotherapy at a later time point, but prior to the transformation. Among all patients with tFL who were systemically treated prior to transformation, 25 (46%) received R-CHOP or R-CVP, 18 (34%) were treated with BR, 4 (7%) received R monotherapy, and 7 (13%) were treated with O-based regimens [4 (7%) with OB and 3 (6%) with O-CHOP].

Maintenance therapy was administered to 50% (n = 335) of all the patients who received chemoimmunotherapy. Among patients with tFL, 37% (n = 20) patients received maintenance therapy prior to transformation: 14 received R and 6 received O (Figure S2 presents patient management prior to transformation and Table S2 presents the characteristics of tFL patients comparing patients who received prior anti-FL chemoimmunotherapy with those who were treatment naïve).

Within the treatment naïve cohort, the most common therapy following transformation was R-CHOP (89%, n = 44). Twelve percent (n = 12) of all patients with tFL underwent autologous stem cell transplantation after transformation.

### 3.5. Time to FL Transformation

The median time from FL diagnosis to transformation was 36 months (range 4–161 months), with estimated transformation rates of 3%, 6.5%, 11.3%, and 13.8% at 2, 5, 10, and 15 years post-diagnosis, respectively (Figure S3 shows the risk of transformation over time). The median time from diagnosis to transformation was not significantly different between treatment-naïve patients (34 months; range 4–158) and previously treated patients (36.8 months; range 4–161; *p* = 0.8).

The median time from initiation of first-line therapy to transformation was 23 months and did not significantly differ between patients receiving upfront therapy (24 months) and those initially managed with observation (21 months; *p* = 0.08). The median number of treatment lines prior to transformation was 1 (range 1–3).

### 3.6. Factors Affecting the Risk of Transformation

Univariate Cox regression analysis did not identify any treatment-related factors significantly associated with HT occurring within 36 months from diagnosis. However, LDH levels showed a trend toward increased risk of transformation (hazard ratio [HR] 2.6; 95% confidence interval [CI], 0.95–7.6; *p* = 0.06).

Among all FL patients, chemoimmunotherapy (whether given upfront or deferred) was not associated with a reduced risk of tFL compared with patients who remained treatment-naïve or received initial radiotherapy. However, when restricting the analysis to patients who received chemoimmunotherapy, deferred treatment (vs. upfront therapy at diagnosis) was associated with an increased risk of HT (HR 1.62, 95% CI 1.06–2.48, *p* = 0.027).

Among all patients who received chemoimmunotherapy for FL, univariate analysis identified several treatment-related factors associated with the risk of transformation (Table S3). Obinutuzumab-based induction regimens vs. R-based regimens were significantly associated with a reduced risk of transformation (HR 0.40, 95% CI 0.18–0.90, *p* = 0.027), as was the administration of any maintenance therapy (HR 0.43, 95% CI 0.25–0.75, *p* = 0.003). Among patients with tFL, transformation appeared to occur earlier in those who were treated with BR, compared to patients who received R-CHOP/R-CVP, with a median time from initiation of treatment to transformation of 16.6 months (IQR 8.07–43.3) versus 25.6 months (IQR 8.5–47.7), respectively, yet it was not statistically significant (*p* = 0.3).

In a multivariate analysis (Table 2), treatment with O-based regimens remained independently associated with a reduced risk of transformation compared to R-based therapy (HR 0.43, 95%CI 0.19–0.97, *p* = 0.042), as was maintenance (with either O or R), which continued to demonstrate a significant protective effect (HR 0.44, 95% CI 0.25–0.78, *p* = 0.005). A sensitivity analysis restricted to patients treated from 2018 onward, when obinutuzumab became available in Israel, yielded similar results (Table S4).

### 3.7. Overall Survival

**Figure 1:**
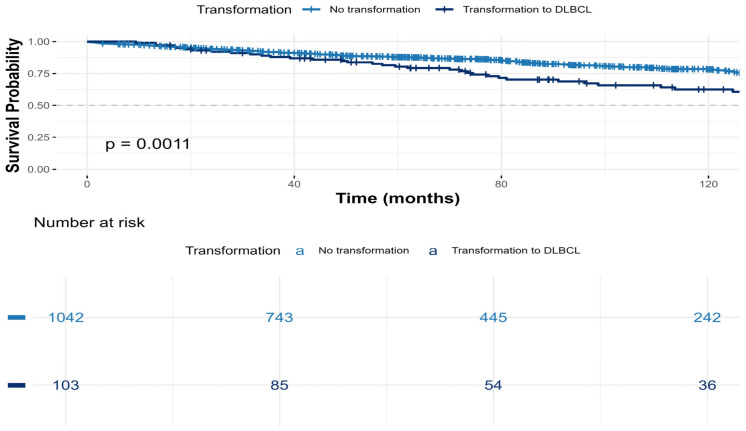
Overall survival of FL patients with and without transformation to DLBCL.

Within a median FU period of 70 months (IQR 36–117), 18% (n = 209) of the patients died: 36% (n = 37) in the tFL cohort and 16.5% (n = 172) in the non-transformed cohort (*p* < 0.001). Overall survival was significantly shorter in patients who developed tFL compared to those who did not (median not reached for both cohorts, *p* = 0.001) (Figure 1).

**Figure 2:**
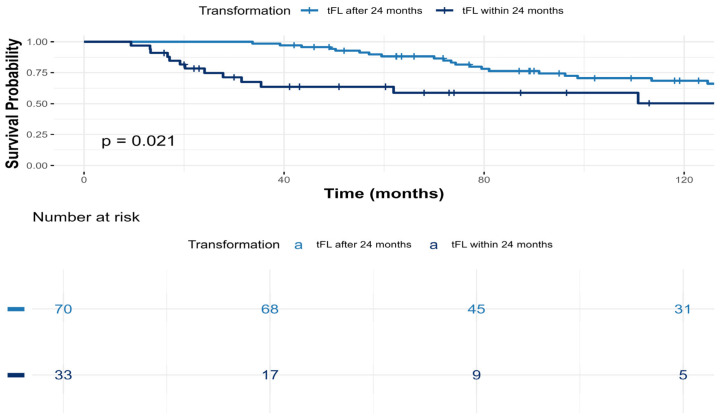
Overall survival of patients with transformed FL by early vs. late transformation.

Among patients with tFL, those who experienced early HT (within 24 months from diagnosis) had inferior survival outcomes, with a 5-year OS after transformation of 65%, compared to 95% among those with later transformation (*p* = 0.02) (Figure 2).

**Figure 3:**
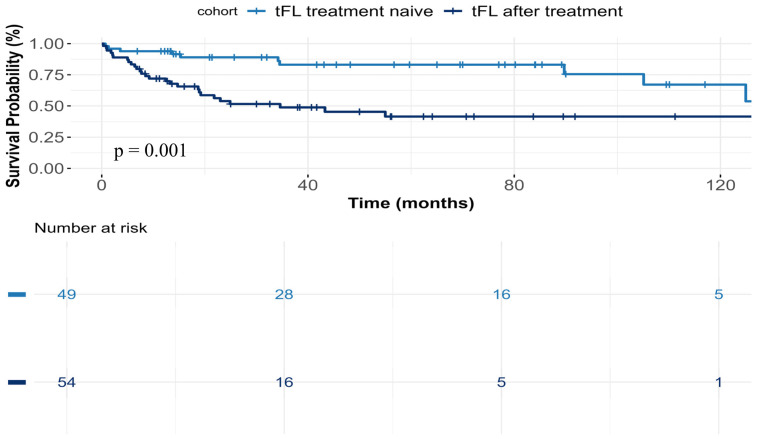
Overall survival of patients with transformed FL by prior chemoimmunotherapy vs. treatment naïve.

Patients who were treatment-naïve at the time of transformation demonstrated significantly longer OS from transformation, compared to those who received prior anti-FL chemoimmunotherapy [median OS not reached vs. 34.6 months, *p* = 0.001 (Figure 3)]. Of note, patients with late transformation were more frequently treatment-naïve at the time of transformation (69%, n = 48).

Patients who had both prior chemoimmunotherapy treatment and early transformation (accounting for 20% (n = 21) of all transformed patients) had a median OS (from the time of transformation) of 18.9 months (IQR 10–31 months) compared to 34.6 months (IQR 16–54 months) in patients with prior chemoimmunotherapy treatment and late transformation (a difference that although impressive, was not statistically significant, *p* = 0.35).

Univariate analysis (Table S5) revealed several clinical variables associated with reduced OS following transformation. These included age over 65 years (HR 2.14, 95% CI 1.05–4.36, *p* = 0.036), male sex (HR 2.14, 95% CI 1.09–4.20, *p* = 0.027), and a CCI score greater than 5 (HR 2.43, 95% CI 1.11–5.36, *p* = 0.027). Pre-existing comorbidities, including NIDDM, HTN, and CVA, were also associated with inferior OS.

Previous exposure to chemoimmunotherapy, compared with no systemic treatment prior to transformation, was a significant predictor of inferior OS (HR 3.61, 95% CI 1.72–7.59, *p* < 0.001). Specifically, patients who received two or more lines of chemoimmunotherapy before transformation had the highest risk for shorter OS (HR 5.84, 95% CI 2.06–16.5, *p* < 0.001) with a median OS post transformation of 24 months (IQR 7.5–29), followed by those who received a single line of systemic therapy (HR 3.3, 95% CI 1.53–7.09, *p* = 0.002), with median OS of 45 months (IQR 8.5–50), compared to treatment-naïve patients (median OS not reached, IQR 15–85).

The specific type of first-line chemoimmunotherapy treatment administered prior to transformation was not significantly associated with OS.

In multivariate analysis (Table 3), factors that remained independently associated with inferior OS among patients with tFL included prior exposure to anti-FL chemoimmunotherapy treatment (HR 5.27, 95%CI 2.36–11.8, *p* < 0.001), male sex (HR 2.36, 95%CI 1.19–4.67, *p* = 0.014), and age over 65 years at the time of transformation (HR 2.54, 95%CI 1.17–5.54, *p* = 0.019).

## 4. Discussion

The transformation of FL to aggressive lymphoma remains a pivotal event in the disease’s natural history, with profound implications for patient outcomes [22]. This large, real-world cohort presents additional insights into the incidence, timing, risk factors, and survival impact of HT, particularly in the context of a variety of first-line therapies and maintenance strategies.

In this study, we provide real-life evidence that O-based induction regimens lower the risk of HT, compared to R, thus corroborating with findings from the GALLIUM trial and expanding their generalizability beyond controlled clinical studies. Additionally, maintenance therapy was associated with lower rates of transformation in our cohort, supporting previous reports that have demonstrated a similar trend [4,5].

These findings are in line with the EuroFollicular Project and the PRIMA trial, which highlighted the protective effect of anti-CD20 antibodies maintenance and the adverse impact of high-risk disease features [7,9]. Our results are further corroborated by a large, pooled European retrospective analysis of over 10,000 patients with follicular lymphoma, which indicated that rituximab, administered during both induction and maintenance phases, significantly reduces the risk of histological transformation [5].

Over a median follow-up of 70 months, we observed a 9% rate of biopsy-proven HT, in a large cohort of patients with FL, which is consistent with rates reported in major population-based and prospective studies in the rituximab era [5,8,9,10,15,23]. This cumulative incidence is comparable to the 5-year transformation rates of 7–15% described in the National LymphoCare Study and by the Spanish Lymphoma Oncology Group, underscoring the ongoing clinical relevance of transformation despite therapeutic advances [8,9].

Notably, the majority of transformations in our cohort occurred relatively early, with a median time to transformation of 36 months from diagnosis of FL, including 32% of the events arising within the first two years from diagnosis. This timing is consistent with real-world registry data from the SEER cohort [15], and with a recent report by Luttwak et al. [24], yet, it is later than the timing reported in the PRIMA [7] and GALLIUM [4] trials, as well as other real-world series, which demonstrated that transformation is most likely to occur within the first 24 months after FL diagnosis [2,4,5,7]. A possible explanation for this discrepancy lies in differences in the compositions of these study populations. Our cohort included a real-world population, comprising patients with both low and high disease burden. In contrast, participants in the PRIMA and Gallium trials were patients with advanced FL, requiring prompt treatment and may have been more likely to present with higher-risk factors, e.g., increased LDH and high Follicular Lymphoma International Prognostic Index (FLIPI) [4,7], predisposing them to earlier transformation. Indeed, the median time from first-line therapy to transformation in previously treated patients in our cohort was 23 months, aligning with the observations from both the PRIMA and GALLIUM trials [4,7].

The high incidence of transformation within the first 24 months from first-line treatment emphasizes the need to identify novel biomarkers that can pinpoint patients at increased risk. It also highlights the importance of vigilant monitoring during this high-risk period, a period already well recognized due to the association between disease progression during that time and poor prognosis (POD24), and the need to investigate interventions that may reduce transformation risk. Such interventions could include selecting specific therapies over others or even considering earlier use of low-toxicity treatments in patients with low-burden disease who are at risk for transformation.

There was no significant difference in median time from first-line therapy to transformation between patients who received upfront chemoimmunotherapy treatment and those who received chemoimmunotherapy treatment after initially managed with observation. This suggests that the risk of transformation likely reflects an underlying disease biology and is not a consequence of prior therapy or its timing [25,26,27]. These findings challenge the notion that exposure to therapy per se accelerates transformation risk, supporting recent data that indicate that transformation can occur independently of treatment history [9,15,22].

Our study confirms several established disease-related risk factors for transformation. Patients with tFL had higher comorbidity burden, as reflected by a higher median Charlson Comorbidity Index (CCI), compared to those who did not have HT, despite similar age and gender distributions. Elevated LDH at diagnosis was associated with a trend toward increased transformation risk (HR 2.6, *p* = 0.06), consistent with prior studies identifying increased LDH as an adverse prognostic marker [4,7,8,9,24]. In line with the existing literature, our data show that among patients who received systemic therapy for FL, upfront observation (watch & wait), with deferred initiation of chemoimmunotherapy, was associated with an increased risk for transformation compared to early initiation of treatment (HR 1.62, *p* = 0.027) [9,12,28,29]. A long-term update of the randomized trial by Northend et al. (2025) [29], which evaluated rituximab monotherapy (with or without maintenance) versus watchful waiting in patients with low-tumor-burden FL, demonstrated no difference in long-term transformation rates between study arms. These findings suggest that anti-CD20 therapy alone does not reduce the risk of transformation in this specific population. However, that trial enrolled exclusively asymptomatic, low-burden patients, in contrast to our real-world cohort, which reflects a broader disease spectrum and included only biopsy-proven transformations.

Treatment-regimen emerged as strong, independent predictors of the risk of transformation. In both univariate and multivariate analyses, O-based regimens were significantly associated with a reduced risk of transformation compared to R-based therapy (HR 0.40–0.43, *p* ≈ 0.025). While it should be noted that the median follow-up period for the O-based cohort was significantly shorter, a factor that warrants caution when comparing long-term outcomes, it is noteworthy that the median time to transformation remained considerably shorter than the total follow-up duration for these patients. This suggests that the observed reduction in transformation risk with O-based therapy remains clinically relevant despite the disparities in follow-up length. Furthermore, a sensitivity analysis restricted to patients treated from 2018 onward, coinciding with the national reimbursement of obinutuzumab in Israel, was associated with a reduced risk of transformation even after adjusting for the treatment era. The GALLIUM trial demonstrated that O-based chemoimmunotherapy significantly reduces the risk of early progression in untreated follicular lymphoma patients, an event frequently associated with HT, with a 34% reduction in the risk of progression, relapse, or death and a 46% reduction in the risk of POD24, compared to R-based therapy [30,31]. Although O regimens were associated with a trend toward reduced risk of transformation in the GALLIUM trial, it was not statistically significant, likely due to incomplete histological confirmation (only 14.6% of patients who had early progression underwent biopsy) [4].

In spite of the potential superiority of O over R in preventing transformation, O has higher toxicity rates (particularly higher rates of infections, cytopenia and second malignancies), marginal progression-free survival benefit, and lack an overall survival advantage [30,32], thus, a selective use of O, guided by new biomarkers predictive of transformation risk, can optimize its therapeutic value.

Transformation to aggressive lymphoma remains a major adverse prognostic event [1,15,33]. In our cohort, OS from FL diagnosis was significantly shorter in patients with tFL (median not reached, *p* < 0.001). Among those with tFL, early transformation (occurring <24 months from diagnosis) was associated with the poorest outcomes, consistent with previous studies showing inferior survival for early versus late transformation [2,5,10,16]. Treatment-naïve patients at the time of transformation had markedly higher survival rates than those previously exposed to anti-FL therapy (median OS not reached vs. 3 years, *p* = 0.001), a finding echoed in multiple studies including the SEER cohorts and the Spanish Lymphoma Oncology Group datasets [6,8,15,34].

In our cohort, treatment-naïve status was frequently associated with late transformation, suggesting that this survival advantage in treatment naïve patients with late transformation is likely to reflect preserved chemosensitivity and intact hematological reserves.

Univariate predictors of inferior survival after transformation included older age, male sex, higher CCI, and prior anti-FL therapy, with the latter remaining independently significant in multivariate analysis (HR 3.27, *p* < 0.001). Notably, the number of prior treatment lines further stratified the risk, with the worst outcomes observed in patients with multiple therapies administered prior to transformation.

This study has several limitations inherent to its retrospective, observational design. First, although the use of a large, real-world cohort enhances generalizability, the reliance on electronic medical records introduces the possibility of incomplete data capture, particularly for treatment details. To minimize potential misclassification of patients with pre-existing DLBCL or FL grade 3B as having early transformation, we excluded cases diagnosed before or within three months of FL diagnosis. This approach may have led to the omission of very early transformation events occurring within this time window, which should be considered when interpreting the results. As only coded information was accessible and narrative text was not available, we were unable to reliably determine treatment indications, FLIPI score, and Positron Emission Tomography-Computed Tomography (PET-CT) findings. The retrospective nature of this study also limits our ability to control for potential confounders, such as unmeasured clinical risk factors. These include the FLIPI score, which is known to predict tFL [7], PET-derived metrics such as Total Metabolic Tumor Volume (TMTV), and genomic profiling [35,36], although the latter is not yet integrated into routine clinical practice. Furthermore, the retrospective design of this study precludes a definitive assessment of how evolving diagnostic standards and longitudinal therapeutic shifts may have influenced the observed outcomes. Temporal variability in treatment patterns, often driven by fluctuating reimbursement policies, represents a potential source of selection or confounding bias. An additional limitation of this study is the difficulty in reliably distinguishing between CHOP- and CVP-based regimens in our database. As anthracycline-containing therapy plays an important role in the treatment of transformed FL, this uncertainty may modestly affect the precision of treatment-related outcomes. Furthermore, the potential for assigning patients with suspected transformation at diagnosis to a CHOP-based regimen cannot be entirely excluded. However, previous reports suggest that CVP was used considerably less often than CHOP in Israel during the study period, which likely minimizes the potential impact of this limitation [32]. Temporal trends in first-line treatment patterns also reflect evolving national treatment and reimbursement policies, with early predominance of R-CHOP/CVP regimens and increasing use of BR- and O-based therapies in recent years. Additionally, maintenance therapy, previously found to be associated with reduced risk for transformation [5], was analyzed as a fixed exposure rather than a time-dependent covariate. We acknowledge that this retrospective approach may introduce a degree of immortal-time bias, as patients must remain transformation-free through the induction phase to qualify for maintenance.

Finally, despite adjustment for known prognostic variables, residual confounding cannot be excluded. Prospective studies with systematic data collection, uniform diagnostic criteria, and longer follow-up are warranted to validate and extend these findings.

## 5. Conclusions

In summary, our real-world analysis confirms that transformation remains a relatively frequent and early event in FL, with the highest risk occurring within the first few years after diagnosis. Among patients who were systemically treated for FL, the risk of transformation was increased in patients who received deferred chemoimmunotherapy treatments compared to those with upfront chemoimmunotherapy treatment.

Our findings suggest that O-based induction and the administration of maintenance regimens are independently associated with a reduced risk of histologic transformation. While these results support a potential protective role for obinutuzumab, they represent a significant association that warrants further prospective validation, particularly given the observational design and a shorter follow-up period in the O-based cohort.

Transformation continues to exert a profound negative impact on survival, particularly in previously treated patients, underscoring the need for individualized, risk-adapted management strategies. These findings provide the first real-world validation of Obinutuzumab’s transformative effect on FL outcomes and support its preferential use in patients at elevated risk of transformation. Future research should focus on the biological mechanisms underlying transformation, optimal surveillance strategies for defining patients at risk for transformation, and refining therapeutic approaches to further mitigate this critical complication.

## Figures and Tables

**Table 1:** Patient characteristics.

Variable	Overall N = 1145	No HT N = 1042	tFL N = 103	*p*-Value
Demographics				
Age at diagnosis, (years), median (range)	61 (19, 93)	61 (19, 93)	63 (23, 87)	0.11
Male, n (%)	543 (47)	494 (47)	49 (48)	>0.9
Socio economic level *, median (IQR)	7.00 (5.00, 9.00)	7.00 (5.00, 9.00)	7.00 (5.00, 9.00)	>0.9
Comorbidities				
NIDDM, n (%)	232 (20)	215 (21)	17 (17)	0.3
HTN, n (%)	434 (38)	391 (38)	43 (42)	0.4
CKD, n (%)	18 (1.6)	17 (1.6)	1 (1.0)	>0.9
Osteoporosis, n (%)	157 (14)	136 (13)	21 (20)	0.039
CCI, median (IQR)	3 (2, 4)	3 (2, 4)	4 (2, 4)	0.007
Treatments				
Time to initiation treatment, months, median (IQR)	3 (1, 17)	3 (1, 13)	10 (2, 40)	<0.001
Treatment strategy, n (%)				0.5
Radiation only	76 (6.6)	68 (6)	8 (8)	
Upfront chemoimmunotherapy	415 (36)	373 (36)	42 (41)	
Upfront observation ^‡^	654 (57)	601 (58)	53 (51)	
Maintenance therapy, n (%)	335 (29)	308 (30)	27 (26)	0.5

Abbreviations: HT, histologic transformation; CCI, Charlson comorbidity index; CKD, Chronic kidney disease; HTN, Hypertension; Non-insulin dependent diabetes mellitus. * Socioeconomic level was derived from an income index categorized into national deciles (1 = lowest income, 10 = highest). ^‡^ Including patients who started therapy > 3 months since diagnosis.

**Table 2:** Multivariate analysis for factors associated with transformation risk among patients who received chemoimmunotherapy.

Variable	HR	95% CI	*p*-Value
Age ≥ 65 years	0.9	0.51, 1.61	0.7
Male	1.33	0.77, 2.28	0.3
Charlson score > 5	1.17	0.47, 2.91	0.7
Obinutuzumab-based regimen	0.43	0.19, 0.97	0.042
Maintenance therapy	0.44	0.25, 0.78	0.005

Abbreviations: CI = Confidence Interval, HR = Hazard Ratio.

**Table 3:** Multivariate analysis for factors associated with shorter OS among patients with transformed FL.

Variable	HR	95% CI	*p*-Value
Age ≥ 65 years	2.54	1.17, 5.54	0.019
Male	2.36	1.19, 4.67	0.014
Charlson score > 5	2.14	0.91, 5.02	0.079
Treated before transformation	5.27	2.36, 11.8	<0.001

Abbreviations: CI = Confidence Interval, HR = Hazard Ratio.

## Data Availability

Individual participant data will not be shared. The data that support the findings of this study are not publicly available due to restrictions imposed by Maccabi Healthcare Services. Access to these data is subject to approval by Maccabi Healthcare Services and is therefore not available from the authors.

## Supplementary Materials

The following supporting information can be downloaded from https://www.mdpi.com/article/10.3390/cancers18091471/s1, Figure S1: Temporal trends in first-line treatment administration during the study period; Figure S2: Patients management in first line (prior to transformation); Figure S3: Cumulative incidence of transformation from FL to DLBCL; Table S1: Patient characteristics comparing rituximab, obinutuzumab and treatment naive; Table S2: Characteristics of patients with transformed follicular lymphoma; Table S3: Univariate analysis for factors associated with transformation risk among patients who received chemoimmunotherapy; Table S4: Sensitivity analysis restricted to the post-2018 era: multivariable analysis of factors associated with transformation risk; Table S5: Univariate analysis for factors associated with shorter OS among patients with transformed FL.

## Abbreviations

The following abbreviations are used in this manuscript:

B2M	Beta-2 microglobulin
BR	Bendamustine + rituximab
CCI	Charlson Comorbidity Index
CHD	Congestive heart disease
CKD	Chronic kidney disease
CI	Confidence interval
CVA	Cerebrovascular accident
DLBCL	Diffuse large B-cell lymphoma
FL	Follicular lymphoma
FLIPI	Follicular Lymphoma International Prognostic Index
FU	Follow-up
Hb	Hemoglobin
HR	Hazard ratio
HT	Histologic transformation
HTN	Hypertension
ICD-9	International Classification of Diseases, Ninth Revision
IQR	Interquartile range
LDH	Lactate dehydrogenase
MHS	Maccabi Healthcare Services
NIDDM	Non-insulin-dependent diabetes mellitus
NLCS	National LymphoCare Study
O	Obinutuzumab
OB	Obinutuzumab + bendamustine
O-CHOP	Obinutuzumab + cyclophosphamide, doxorubicin, vincristine, prednisone
O-CVP	Obinutuzumab + cyclophosphamide, vincristine, prednisone
OS	Overall survival
PET-CT	Positron Emission Tomography–Computed Tomography
R	Rituximab
R-CHOP	Rituximab + cyclophosphamide, doxorubicin, vincristine, prednisone
R-CVP	Rituximab + cyclophosphamide, vincristine, prednisone
tFL	Transformed follicular lymphoma
