# Supplementary material for: Histologic Transformation in Follicular Lymphoma: Real-World Outcomes with Rituximab vs. Obinutuzumab-Based Combinations
Source: Cancers (Basel). 2026 May 3;18(9):1471. doi: 10.3390/cancers18091471 (PMC13162783; doi:10.3390/cancers18091471)

Histologic Transformation in Follicular Lymphoma: Real-World Outcomes with Rituximab vs Obinutuzumab-Based Combinations

Supplementary tables & figures

Table S1. Patient characteristics comparing rituximab, obunituzumab and treatment naive

Variable	**Overall** N = 1,096	**Rituximab based regimens** N=433	**Obinutuzumab based regimens** N=187	**Treatment naïve** N=476	**p-value**
**Demographics**					
Age at diagnosis, (years), median (range)	61 (51, 70)	62 (52, 70)	59 (47, 66)	61 (51, 72)	<0.001
Male, n(%)	522 (48%)	190 (44%)	105 (56%)	227 (48%)	0.019
Socio economic level, median (IQR)	7.00 (5.00, 9.00)	7.00 (5.00, 9.00)	7.00 (5.00, 9.00)	7.00 (5.00, 9.00)	0.7
**Comorbidities**					
NIDDM, n(%)	229 (21%)	97 (22%)	37 (20%)	95 (20%)	0.6
Osteoporosis, n(%)	146 (13%)	64 (15%)	11 (5.9%)	71 (15%)	0.004
CKD, n(%)	18 (1.6%)	7 (1.6%)	2 (1.1%)	9 (1.9%)	0.8
CVA, n(%)	11 (1.0%)	6 (1.4%)	0 (0%)	5 (1.1%)	0.3
HTN, n(%)	415 (38%)	180 (42%)	56 (30%)	179 (38%)	0.023
Dementia, n(%)	16 (1.5%)	5 (1.2%)	1 (0.5%)	10 (2.1%)	0.3
CHF, n(%)	20 (1.8%)	11 (2.5%)	0 (0%)	9 (1.9%)	0.067
**FL-related lab results**					
LDH > 280 U/L, n(%)	637 (78%)	280 (82%)	117 (77%)	240 (75%)	0.057
Hb < 12g/dL, n(%)	186 (18%)	93 (22%)	29 (16%)	64 (15%)	0.011
B2M>3mg/L, n(%)	140 (50%)	70 (61%)	21 (48%)	49 (41%)	0.007
Treatment strategy, n(%)					
Radiation only	72 (6.6%)	15 (3.5%)	8 (4.3%)	49 (10%)	
Upfront chemoimmunotherapy	409 (37%)	287 (66%)	122 (65%)	0 (0%)	
Upfront observation	615 (56%)	131 (30%)	57 (30%)	0 (%)	

Table S1. Patient characteristics comparing rituximab, obunituzumab and treatment naive					
Maintenance therapy	328 (30%)	207 (48%)	121 (65%)	0 (0%)	<0.001
**Follow up time (months), median(IQR)**	68 (35, 114)	86 (48, 124)	48 (31, 70)	67 (29, 124)	<0.001
**Status at the end of follow-up, n(%)**					
Alive	882 (80%)	333 (77%)	169 (90%)	380 (80%)	
Death	199 (18%)	94 (22%)	18 (9.6%)	87 (18%)	
Censored	15 (1.4%)	6 (1.4%)	0 (0%)	9 (1.9%)	

Abbreviations: β2M, Beta-2 microglobulin; CHF, Congestive heart failure; CKD, Chronic kidney disease; CVA, Cerebrovascular accident; Hb, Hemoglobin; HTN, Hypertension; LDH, Lactate dehydrogenase; NIDDM, Non-insulin dependent diabetes mellitus.

Table S2. Characteristics of patients with transformed follicular lymphoma				
Variable	**Overall** N = 103[1]	**Treatment naïve** N = 49	**Treatment before tFL** N = 54	**p-value**
**Demographics**				
Age at diagnosis, years, median (IQR)	63 (56, 69)	64 (57, 69)	61 (54, 69)	0.2
Male, n(%)	49 (48)	21 (43)	28 (52)	0.4
Socio economic level, median (IQR)*	7.00 (5.00, 9.00)	8.00 (6.00, 9.00)	7.00 (5.00, 9.00)	0.4
**Comorbidities**				
NIDDM, n(%)	17 (17)	3 (6.1)	14 (26)	0.007
Osteoporosis, n(%)	21 (20)	11 (22)	10 (19)	0.6
CKD, n(%)	1 (1.0)	0 (0)	1 (1.9)	>0.9
CVA, n(%)	3 (2.9)	1 (2.0)	2 (3.7)	>0.9
HTN, n(%	43 (42)	19 (39)	24 (44)	0.6
CHF, n(%)	1 (1.0)	0 (0)	1 (1.9)	>0.9
**FL-related lab results, n(%)**				
LDH > 280 U/L†	62 (81)	30 (77)	32 (84)	0.4
Hb < 12g/dL	18 (18)	4 (9)	14 (27)	0.02
B2M>3mg/L, n(%)	19 (66)	6 (43)	13 (87)	0.021
**Treatment strategy, n(%)**				<0.001
Radiation only	8 (7.8)	4 (8.2)	4 (7.4)	
Upfront therapy	42 (41)	6 (12)	36 (67)	
Upfront observation‡	53 (51)	39 (80)	14 (26)	
Time to transformation, months, median (IQR)	36 (16, 79)	34 (12, 82)	37 (18, 72)	0.9
2nd line before transformation, n(%)	9 (8.7)	0 (0)	9 (17)	0.003
Follow up time since diagnosis (months), median(IQR)	87 (50, 150)	114 (66, 157)	72 (42, 118)	0.011
**Status at the end of follow-up, n(%)**				0.003
Alive	65 (63)	38 (78)	27 (50)	
Death	37 (36)	10 (20)	27 (50)	

Table S2. Characteristics of patients with transformed follicular lymphoma				
Variable	**Overall**<br>N = 103[1]	**Treatment naïve**<br>N = 49	**Treatment before tFL**<br>N = 54	**p-value**
Censored[§]	1 (1.0)	1 (2.0)	0 (0)	

Abbreviations: β2M, Beta-2 microglobulin; CHF, Congestive heart failure; CKD, Chronic kidney disease; CVA, Cerebrovascular accident; Hb, Hemoglobin; HTN, Hypertension; LDH, Lactate dehydrogenase; NIDDM, Non-insulin dependent diabetes mellitus.

* Socioeconomic level was derived from an income index categorized into national deciles (1=lowest income, 10=highest).

† Elevated LDH defined as >280 U/L, the upper limit of normal in MHS laboratories.

‡ Including patients that started therapy >3 months since diagnosis

§ Censored = patients who left MHS before the end of follow-up and were not active members at the time of data extraction.

Table S3.Univariate analysis for factors associated with transformation risk among patients who received chemoimmunotherapy

Variable	HR	95% CI	p-value
Age at diagnosis, years	1.01	0.99, 1.03	0.3
Age≥65 years	1.14	0.67, 1.95	0.6
Male	1.21	0.71, 2.07	0.5
Socio economic level*	0.98	0.87, 1.11	0.8
NIDDM	1.33	0.72, 2.45	0.4
Osteoporosis	1.79	0.90, 3.56	0.1
CKD	1.24	0.17, 8.96	0.8
HTN	1.32	0.77, 2.26	0.3
CHF	1.04	0.14, 7.54	>0.9
CCI > 5	1.31	0.56, 3.07	0.5
LDH > 280 U/L†	0.95	0.4, 2.28	>0.9
Hb < 12g/dL	1.38	0.75, 2.54	0.3
B2M>3mg/L	3.95	0.89, 17.5	0.07
Bendamustine based regimen	0.65	0.37,1.12	0.12
Obinutuzumab based regimen	0.40	0.18, 0.90	0.027
Rituximab based regimen	2.48	1.11, 5.55	0.027
Maintenance therapy	0.43	0.25, 0.75	0.003

Abbreviations: β2M, Beta-2 microglobulin; CCI, Charlson comorbidity index; CHF, Congestive heart failure; CKD, Chronic kidney disease; CVA, Cerebrovascular accident; Hb, Hemoglobin; HTN, Hypertension; LDH, Lactate dehydrogenase; NIDDM, Non-insulin dependent diabetes mellitus.

* Socioeconomic level was derived from an income index categorized into national deciles (1=lowest income, 10=highest).

† Elevated LDH defined as >280 U/L, the upper limit of normal in MHS laboratories.

Table S4. Sensitivity analysis restricted to the post-2018 era: multivariable analysis of factors associated with transformation risk

Variable	HR	95% CI	p-value
Age≥65 years	0.94	0.39, 2.2	0.9
Male	1.52	0.66, 3.51	0.3
Charlson score > 5	1.03	0.7, 3.2	0.7
Obinutuzumab based regimen	0.36	0.23, 0.95	0.008
Maintenance therapy	0.54	0.39, 0.84	0.01

Abbreviations: CI = Confidence Interval, HR = Hazard Ratio

Table S5.Univariate analysis for factors associated with shorter OS among patients with transformed FL

**Variable**	**HR**	**95% CI**	**p-value**
Age at diagnosis, years	1.04	1.00, 1.07	0.026
Age≥65 years	2.14	1.05, 4.36	0.036
Male	2.14	1.09, 4.20	0.027
Socio economic level*	1.03	0.89, 1.19	0.7
NIDDM	2.64	1.23, 5.68	0.013
Osteoporosis	1.62	0.78, 3.36	0.2
CKD	6.76	0.89, 51.4	0.065
CVA	4.33	1.31, 14.3	0.016
HTN	3.13	1.59, 6.17	0.001
CCI > 5	2.43	1.11, 5.36	0.027
Treatment before transformation	3.61	1.72, 7.59	<0.001
Number of treatment lines before transformation			
1	—	—	
2	3.95	1.65, 9.43	0.002
3	7.01	2.29, 21.5	<0.001
Obinutuzumab based induction therapy	1.24	0.38, 4.06	0.7
Rituximab based induction regimen	0.92	0.38, 2.21	0.8

Abbreviations: CCI, Charlson comorbidity index; CHF, Congestive heart failure; CKD, Chronic kidney disease; CVA, Cerebrovascular accident; Hb, Hemoglobin; HTN, Hypertension; LDH, Lactate dehydrogenase; NIDDM, Non-insulin dependent diabetes mellitus.

* Socioeconomic level was derived from an income index categorized into national deciles (1=lowest income, 10=highest).

Figure S1. Temporal trends in first-line treatment administration during the study period. The table below presents the absolute numbers and proportions of each treatment regimen across the defined time periods.

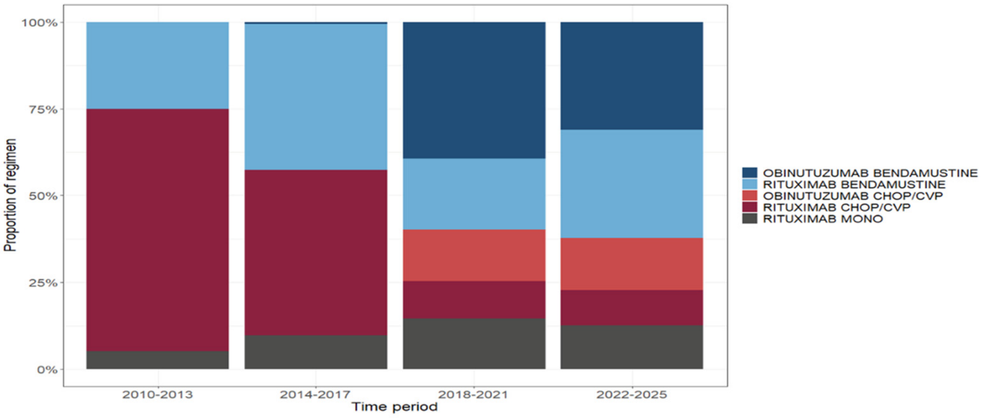

Regimen	Overall N=620	2010-2013 N=96	2014-2017 N=164	2018-2021 N=241	2022-2024 N=119
OB, n(%)	133 (21)	0 (0)	1 (0.2)	95 (39)	37 (31)
RB, n(%)	179 (29	24 (25)	69 (42)	49 (20)	37 (31)
O-CHOP/CVP, n(%)	54 (9)	0 (0)	0 (o)	36 (15)	18 (15)
R-CHOP/CVP, n(%)	183 (30)	67 (70)	78 (48)	26 (11)	12 (10)
R-monotherapy, n(%)	71 (11)	5 (5)	16 (9.8)	35 (15)	15 (13)

Abbreviations: O-CHOP/CVP, Obinutuzumab combined with cyclophosphamide, doxorubicin, vincristine and prednisone or cyclophosphamide, vincristine and prednisone; OB, Obinutuzumab plus bendamustine; R-CHOP/CVP, Rituximab with CHOP/CVP; RB, Rituximab plus bendamustine; R-MONO, Rituximab monotherapy

Figure S2 - Patients management in first line (prior to transformation).

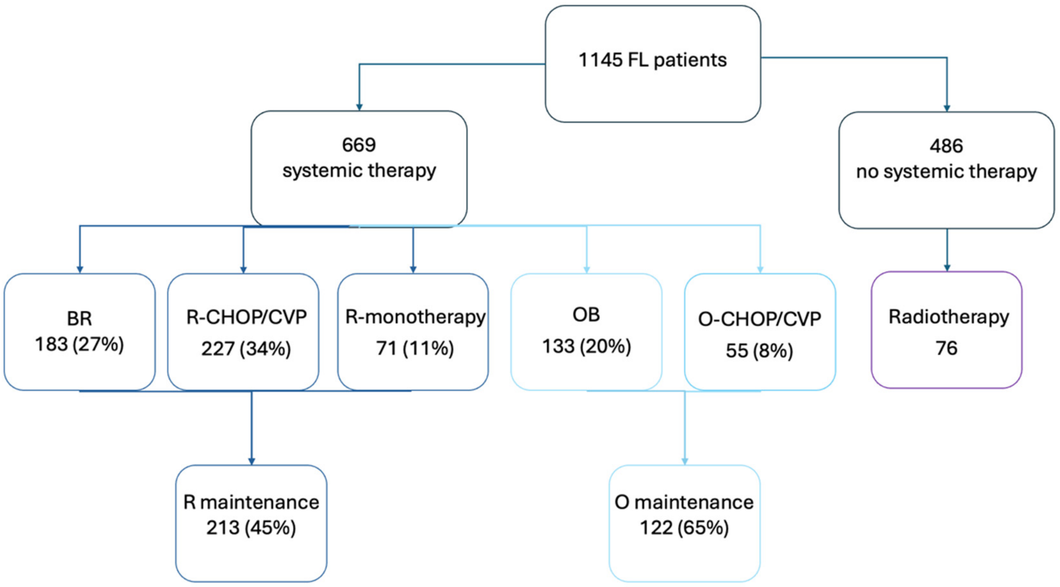

Figure S3- Cumulative incidence of transformation from FL to DLBCL- The Kaplan-Meier shows transformation risk over time. The median time from follicular lymphoma diagnosis to transformation was 36 months (range, 4–161 months). The estimated cumulative probability of transformation was 3.0%, 6.5%, 11.3%, and 13.8% at 2, 5, 10, and 15 years following diagnosis, respectively.

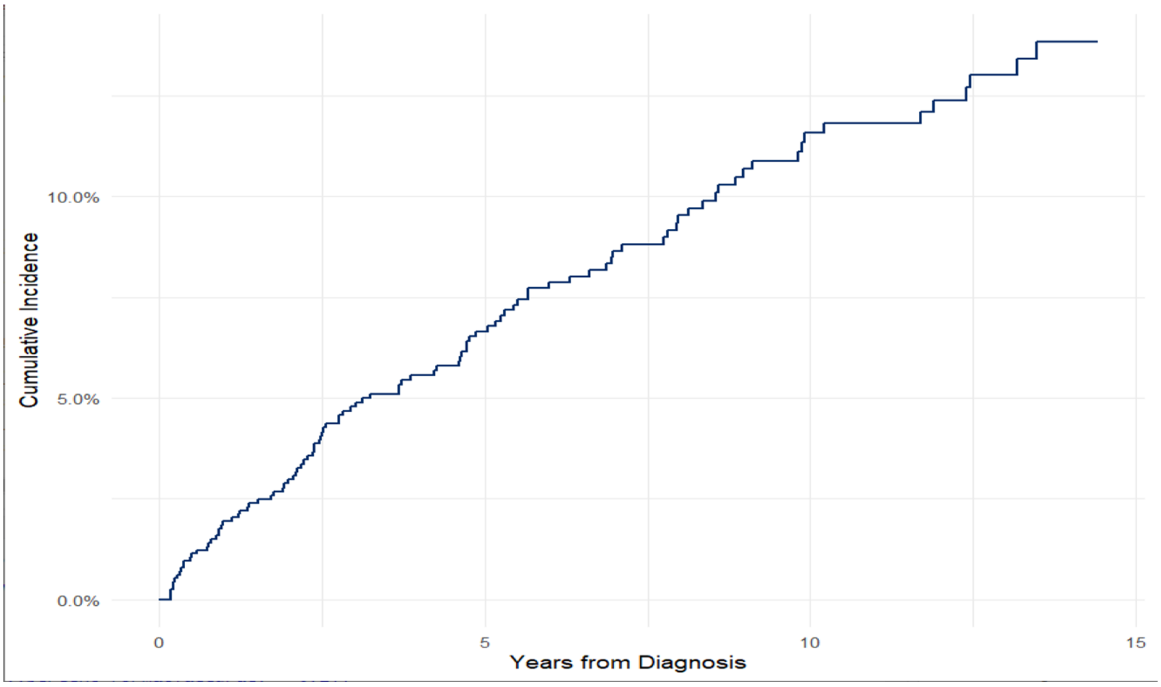

Supplement: Supplementary file 1 [file cancers-18-01471-s001.zip › cancers-4260308-supplementary.pdf]
